# Exposure to Nanoscale Particulate Matter from Gestation to Adulthood Impairs Metabolic Homeostasis in Mice

**DOI:** 10.1038/s41598-018-37704-2

**Published:** 2019-02-12

**Authors:** Nicholas C. Woodward, Amanda L. Crow, Yang Zhang, Sam Epstein, Jaana Hartiala, Richard Johnson, Heidi Kocalis, Arian Saffari, Ishwarya Sankaranarayanan, Omid Akbari, Gajalakshmi Ramanathan, Jesus A. Araujo, Caleb E. Finch, Sebastien G. Bouret, Constantinos Sioutas, Todd E. Morgan, Hooman Allayee

**Affiliations:** 1 Departments of Preventive Medicine and Biochemistry & Molecular Medicine, Keck School of Medicine, University of Southern California, Los Angeles, CA 90033 USA; 2 Leonard Davis School of Gerontology, University of Southern California, Los Angeles, California 90089 USA; 3 The Saban Research Institute, Developmental Neuroscience Program and Diabetes and Obesity Program, Center for Endocrinology, Diabetes and Metabolism, Children’s Hospital Los Angeles, University of Southern California, Los Angeles, CA 90027 USA; 4 Inserm, Jean-Pierre Aubert Research Center, U1172, University Lille 2, Lille, 59045 France; 5 Department of Civil and Environmental Engineering, Viterbi School of Engineering, University of Southern California, Los Angeles, California 90089 USA; 6 Department of Molecular Microbiology and Immunology, Keck School of Medicine, University of Southern California, Los Angeles, CA 90033 USA; 7 Department of Medicine, David Geffen School of Medicine at UCLA, Los Angeles, CA 90095 USA; 8 Department of Environmental Health Sciences, Fielding School of Public Health at UCLA, Los Angeles, CA 90095 USA

## Abstract

Emerging evidence from epidemiological and animal studies suggests that exposure to traffic-related air pollutants and particulate matter less than 2.5 µm in diameter (PM_2.5_) contributes to development of obesity and related metabolic abnormalities. However, it is not known whether nanoscale particulate matter (nPM) with aerodynamic diameter ≤200 nm have similar adverse metabolic effects. The goal of the present study was to determine the effects of prenatal and early life exposure to nPM on metabolic homeostasis in mice. C57BL/6 J mice were exposed to nPM or filtered air from gestation until 17 weeks of age and characterized for metabolic and behavioral parameters. In male mice, nPM exposure increased food intake, body weight, fat mass, adiposity, and whole-body glucose intolerance (p < 0.05). Consistent with these effects, male mice exposed to nPM displayed alterations in the expression of metabolically-relevant neuropeptides in the hypothalamus and decreased expression of insulin receptor signaling genes in adipose (p < 0.05). There were no differences in exploratory behavior or motor function, fasting lipid levels, or the inflammatory profile of adipose tissue. Our results provide evidence that chronic nPM exposure from gestation to early adulthood in male mice promotes metabolic dysregulation in part through modulation of feeding behavior and in the absence of an obesogenic diet.

## Introduction

It is generally accepted that obesity is characterized by lifetime exposure to an obesogenic environment in the context of genetic susceptibility factors. In this regard, prior studies have focused on the imbalance between caloric intake and energy expenditure as one root environmental cause for the increased prevalence of obesity. However, emerging data from human and animal studies suggest that exposure to air pollution during *in utero*, early life, and later developmental periods may also play a role in the development of obesity and related metabolic abnormalities^1^. For example, epidemiological studies have shown that prenatal and childhood exposure to ambient or traffic-related air pollutants (TRAP) is associated with more rapid BMI increases during childhood, increased prevalence of obesity, and metabolic dysregulation^2–8^. In adults, similar associations have been reported with higher incidence of metabolic syndrome, insulin resistance, and diabetes^9–17^. Collectively, these observations suggest that exposure to ambient air pollution, including during critical periods of development, may contribute to obesity in early life and its metabolic consequences later in adulthood.

The epidemiological data linking TRAP and obesity in humans has garnered considerable interest in the use of animal models to identify potential underlying pathophysiological mechanisms^18,19^. For the most part, mouse studies focused on obesity have evaluated the effects of regional fine particulate matter, defined less than 2.5 µm in diameter (PM_2.5_), with or without high fat feeding. These studies have shown that exposure to PM_2.5_ modulates adiposity, particularly with respect to visceral fat accumulation, the development of glucose intolerance and other related metabolic abnormalities^20–22^. Furthermore, *in utero* exposure to diesel exhaust particles (DEP), another model of TRAP, increased fetal brain inflammatory cytokines and, in conjunction with a high fat diet, led to microglial activation and increased anxiety in adulthood^23^. While the mechanisms for these adverse effects are not entirely known, these studies collectively suggest that ambient air pollution exposure impairs metabolic homeostasis through alterations of biological processes in the periphery as well as the central nervous system.

Within the size spectrum of PM_2.5_, nanoscale particulate matter (nPM) with aerodynamic diameter ≤200 nm, which are emitted primarily through vehicular emissions and other combustion sources, may also be of particular relevance to obesity and metabolic health. For example, these nanoscale particles contain a high content of redox-cycling organic chemicals and can have higher biological activity than larger particulates due to their higher surface area-to-mass ratio^24^. While nPM exposure in mice during gestation or adulthood has been linked to impaired neuronal differentiation and increased microglial activation^25–27^, no studies have directly examined whether these particles influence obesity-related outcomes. Therefore, the goals of this study were to determine the effects of nPM exposure on metabolic homeostasis in mice.

## Results

### Body weight and composition

**Figure 1:**
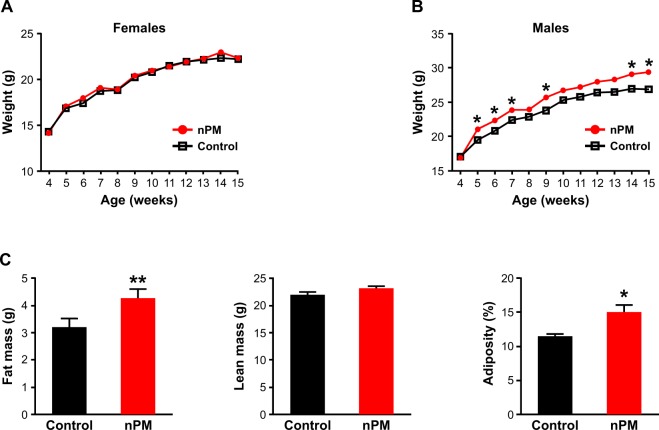
Sex-specific effects of nPM exposure on body weight and composition. Female mice exposed to nPM did not have differences in body weight compared to or control mice exposed to filtered air between 5–15 weeks of age. (**A**) Starting at 5 weeks of age, male mice exposed to nPM had significantly higher body weight at various time points up to 15 weeks of age compared to control animals. (**B**) Whole body composition analysis by NMR showed that 17-week old male mice exposed to nPM had significantly increased fat mass, but not lean mass, and increased adiposity. (**C**) Data are shown as mean ± SE from 6–9 mice per group. Control and nPM groups are indicated by black and red bars, respectively. *p < 0.05; **p < 0.01.

As a first step towards characterizing the effects of ultrafine particulate matter on metabolic homeostasis in mice, we carried out an exposure protocol starting at gestation and continuing through young adulthood. At weaning, body weight in female and male mice exposed to nPM (Fig. 1A,B) was not significantly different from the control groups. Female body weights remained unchanged by nPM exposure throughout the experiment (Fig. 1A). By comparison, male mice exposed to nPM exhibited a pattern of significantly increased body weight compared to age-matched control mice starting between 5–7 weeks of age and at older ages, including a 10% increased body weight at the end of the exposure period (Fig. 1B). Given these sex-specific effects on body weight and similar findings reported in previous studies with DEP exposure^28^, we focused our efforts on further characterizing male mice. At the end of the exposure period, 17-week old nPM-exposed male mice had significantly greater total fat mass, but not lean body mass, and ~30% increased adiposity compared to control mice (Fig. 1C and Table 1). These results suggest that the higher body weight observed in male mice exposed to nPM was primarily due to increased accumulation of adipose tissue.

**Table 1:** Metabolic Characteristics of Male Mice Exposed to nPM or Filtered Air.

Trait	Control	nPM	p-value
Body weight (g)	27.9 ± 0.6	29.6 ± 0.6	0.045
Lean Mass (g)	22.1 ± 0.5	23.0 ± 0.5	0.12
Fat mass (g)	3.2 ± 0.3	4.3 ± 0.3	0.009
Adiposity (%)	11.6 ± 1.1	14.9 ± 0.9	0.02
Glucose (mg/dL)	134 ± 11	134 ± 7	0.50
Insulin (pg/mL)	433 ± 83	367 ± 50	0.26
Leptin (ng/mL)	1498 ± 618	1637 ± 398	0.43
Triglycerides (mg/dL)	20 ± 2	21 ± 2	0.45
Total cholesterol (mg/dL)	89 ± 6	84 ± 4	0.26
HDL cholesterol (mg/dL)	61 ± 3	62 ± 3	0.43
VLDL/LDL cholesterol (mg/dL)	28 ± 4	23 ± 2	0.11
Liver triglyceride content (μg/mg protein)	64.4 ± 8.8	72.1 ± 14.3	0.33
Liver cholesterol content (μg/mg protein)	8.7 ± 1.4	6.3 ± 0.5	0.07

Data are from 6–7 mice in each group and shown as mean ± SE.

HDL, high-density lipoprotein; VLDL, very-density lipoprotein; LDL, low-density lipoprotein.

### Metabolic and behavioral parameters

**Figure 2:**
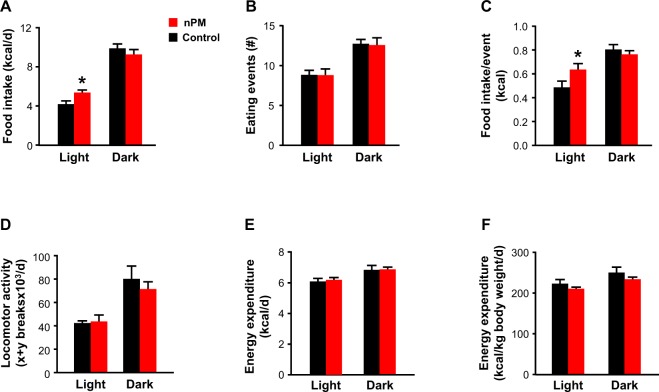
Effect of nPM exposure on feeding behavior locomotor activity, and energy expenditure. Male mice exposed to nPM exhibited significantly increased food intake during the light cycle, but not dark cycle, compared to control mice. (**A**) Increased food intake was not due to an increased number of eating events (**B**) but the number of kcals consumed per event. (**C**) There were no differences in total locomotor activity (**D**) or energy expenditure, expressed as either per day (**E**) or normalized body weight. (**F**) Data are shown as mean ± SE from 4 mice in each exposure group. Control and nPM groups are indicated by black and red bars, respectively. *p < 0.05.

To determine whether changes in energy balance could explain the differences in body weight and composition, 17-week old male mice were placed in metabolic cages and feeding behavior, locomotor activity, and energy expenditure were continuously monitored over 4 consecutive days. Food consumption was 28% higher in nPM-exposed male mice compared to controls (5.4 ± 0.2 kcal/day vs. 4.2 ± 0.3 kcal/day; p = 0.01) but this difference was only due to increase food intake during the light cycle (Fig. 2A). Furthermore, although the total number of feeding events was unchanged (Fig. 2B), nPM exposed mice consumed 30% more kcal per event (Fig. 2C, p < 0.05). By comparison, locomotor activity, energy expenditure in either the light or dark cycles (Fig. 2D–F), and VCO_2_, VO_2_, and respiratory exchange ratio (RER) (Supplementary Fig. 1) were not affected by nPM exposure.

We next tested whether the effects of nPM exposure on obesity-related traits could be due to changes in exploratory behavior and overall motor function. Performance in open field tests for velocity, total distance traveled, or percent time spent in the periphery was not significantly different as a function of nPM exposure (Supplementary Fig. 2). Rotarod learning and performance in 4 RPM, 16 RPM, and acceleration protocols, were also unchanged suggesting minimal impact of nPM exposure on physical capacity or lung function (Supplementary Fig. 2). Other behavioral traits, such as working and recognition memory or anxiety, were not different either between nPM-exposed and control mice (Supplementary Fig. 3). At the tissue level, concentrations of cortical serotonin, noradrenaline, and dopamine or metabolites of these neurotransmitters were similarly unaffected by nPM exposure (Supplementary Fig. 3). Taken together, these data indicate that altered body weight and composition as a result of nPM exposure could be due, in part, to increased food intake but not other parameters related to energy balance, exploratory behavior, and motor function.

### Physiological traits and glucose tolerance

**Figure 3:**
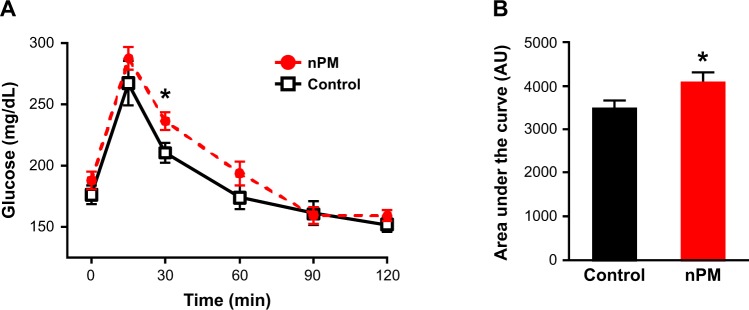
Effect of nPM exposure on peripheral glucose tolerance. Intra-peritoneal glucose tolerance tests (IPGTTs) demonstrate that plasma glucose levels 30 minutes after a bolus glucose injection were significantly higher in nPM-exposed male mice (filled red circles) compared to controls (open squares). The overall glucose tolerance profile, as calculated by the area under of the curve, was also significantly worse in nPM-exposed mice than controls. (**B**) IPGTT experiments were performed as described in the Materials and Methods section. Data are shown as mean ± SE from 6–7 mice in each exposure group. Control and nPM groups are indicated by black and red bars, respectively. *p < 0.05.

We next determined whether the increased adiposity by nPM exposure led to other metabolic disturbances. At 16 weeks of age, fasting glucose at the baseline timepoint of intraperitoneal glucose tolerance tests (IPGTTs) was not affected by nPM exposure (Fig. 3A**)**. However, nPM-exposed male mice had 15% increased plasma glucose levels 30 minutes after the bolus glucose injection (Fig. 3A, p < 0.05). In addition, the overall glucose disposal profile of male mice exposed to nPM was increased 17% compared to control mice, as illustrated by higher area under the curve (Fig. 3B). Lastly, nPM exposure did not alter fasting plasma insulin, leptin, or lipid levels, or hepatic lipid content (Table 1).

### Gene expression and cellular profiles of metabolic tissues

**Figure 4:**
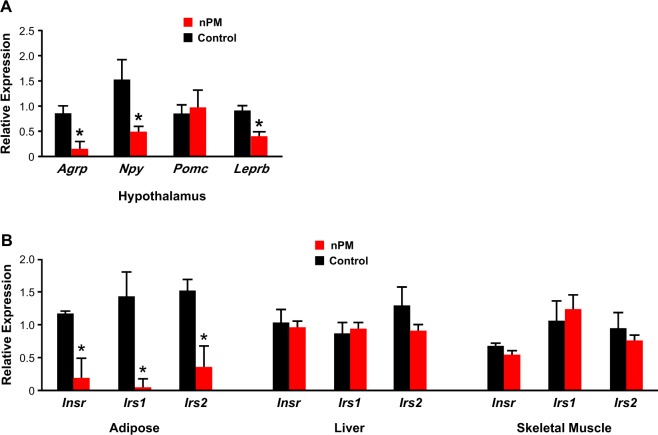
Effect of nPM exposure on gene expression in metabolic tissues. Exposure to nPM led to significantly lower expression of agouti-related protein (*Agrp*), neuropeptide Y (*Npy*) and the long isoform of the leptin receptor (*Leprb*) in the hypothalamus of male mice compared to controls. (**A**) The mRNA levels of insulin receptor (*Insr*) and insulin receptor substrates 1 and 2 (*Irs1* and *Irs2*) were significantly decreased in adipose tissue, but not liver or skeletal muscle, of nPM-exposed male mice compared to controls. (**B**) Gene expression analysis was carried out by real-time quantitative PCR in quadruplicate with SYBR green assays. RNA levels for each sample were normalized to *Ppia* or *Gapdh*, as endogenous controls, and the replicates were averaged to determine differences between control and nPM exposure. Data are shown as mean ± SE from 5–7 mice in each exposure group. Control and nPM groups are indicated by black and red bars, respectively. *p < 0.05.

**Figure 5:**
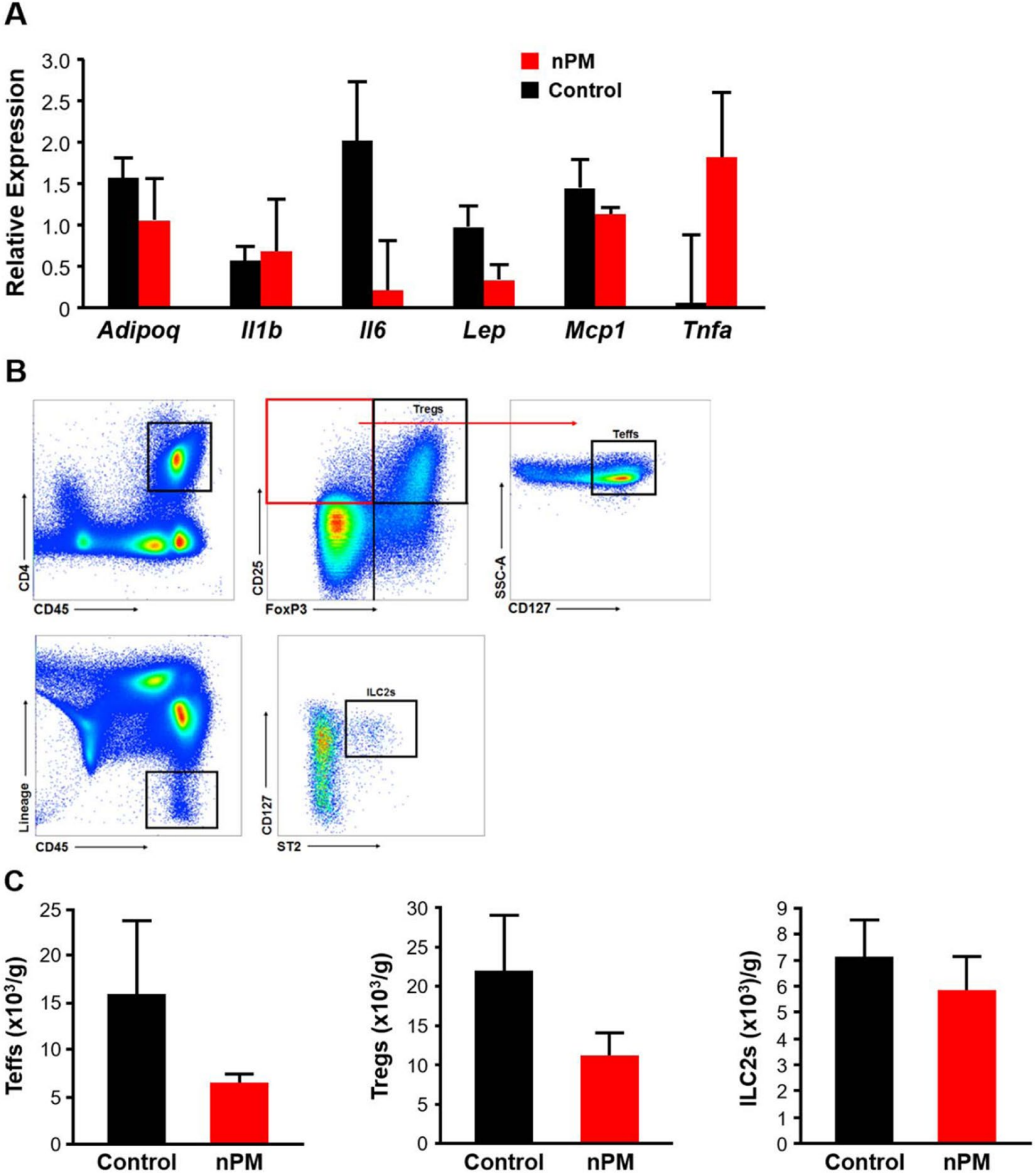
Effect of nPM exposure on inflammatory gene expression and immune cell profiles in adipose tissue. Expression levels of various adipocytokines were not significantly different between nPM-exposed male mice compared to controls. Gene expression analysis was carried out by real-time quantitative PCR in quadruplicate with SYBR green assays. RNA levels for each sample were normalized to *Ppia* or *Gapdh*, as endogenous controls, and the replicates were averaged to determine differences between control and nPM exposure. (**A**) Representative flow cytometry plots for the gating strategy used to quantify numbers of regulatory T cells (Tregs), effector T cells (Teffs), and type 2 innate lymphoid cells (ILC2s). (**B**) The number of Tregs, Teffs, and ILC2s were not significantly different between male mice exposed to nPM compared to controls. (**C**) Data are shown as mean ± SE from 4–5 mice for gene expression analyses and from 3–4 mice for flow cytometric analyses in adipose tissue. Control and nPM groups are indicated by black and red bars, respectively. *p < 0.05.

To gain insight into how nPM exposure increased food intake and glucose intolerance, we carried out gene expression analysis in metabolically relevant tissues in 18-week old mice. In the hypothalamus, nPM-exposed mice had significantly decreased expression of genes involved in appetite regulation, such as *Agrp* (80% decrease), *Npy* (−70%), and *Leprb* (−55%) (Fig. 4A). Furthermore, mRNA levels of genes that participate in receptor-mediated insulin signaling, such as *Inrs*, *Irs1*, and *Irs2*, were significantly decreased by 85%, 95%, and 75%, respectively, in adipose tissue of nPM-exposed mice compared to controls (Fig. 4B**)**. By comparison, *Inrs*, *Irs1*, and *Irs2* expression was not altered in liver or skeletal muscle (Fig. 4B). We next determined whether the increased adiposity and decreased expression of insulin signaling genes in nPM-exposed mice was associated with a pro-inflammatory profile in adipose tissue. However, there were no differences in mRNA levels of genes encoding various adipocytokines involved in obesity and insulin resistance (Fig. 5A). Lastly, flow cytometric analysis of adipose tissue revealed non-significant decreases in the number of T effector cells (Teffs) and T regulatory cells (Tregs) but no changes in type 2 innate lymphoid cells (ILC2s) (Fig. 5B,C).

## Discussion

Using an exposure protocol initiated at conception and extending to the equivalent of young adulthood in humans, the metabolic effects of nPM first became evident at 5 weeks of age where exposed male, but not female, mice exhibited increased body weight. These sex-specific effects persisted until the mice were euthanized at 18 weeks of age, at which time nPM-exposed male mice had mild obesity associated with hyperphagia but with similar energy expenditure and activity levels. Consistent with these observations, nPM exposure impaired whole-body glucose tolerance and decreased expression of insulin signaling genes in adipose tissue but not liver or skeletal muscle. Notably, the obesity-related effects in this study occurred without high fat feeding, suggesting that nPM exposure alone, at least in the context of our relatively short-term study, was sufficient to cause the observed physiological abnormalities. Despite the increased adiposity and impaired glucose tolerance, gene expression or immune cell composition analyses of adipose tissue did not reveal an increased inflammatory profile that is often associated with obesity^29^. Interestingly, prolonged exposure to PM_2.5_ for 10 months has been shown to induce glucose intolerance and increased inflammation in visceral fat depots in the absence of a high fat diet^21^. It is possible that the duration and/or amount of nPM exposure we used was insufficient to promote inflammation in adipose tissue, particularly since the adiposity effects we observed were relatively modest compared to diet-induced obesity in C57Bl/6 mice^30^. By comparison, we previously demonstrated that nPM exposure in the context of a high fat diet and genetic hyperlipidemia led to a pro-atherogenic lipoprotein phenotype and increased aortic lesion formation^31,32^. Thus, additional studies will be required to determine whether an obesogenic diet, higher particle concentrations, and/or prolonged exposure times are necessary in order for nPM to induce inflammation in adipose and further exacerbate metabolic dysregulation.

Accumulating evidence suggest that various outdoor air pollutants, such as particulate matter, polycyclic aromatic hydrocarbons, black carbon, heavy metals, and ozone, can have adverse effects on the nervous system^33^. In this regard, nPM exposure has been shown to influence the central nervous system through either direct or indirect mechanisms^34–36^, including increased microgial-mediated neuroinflammation in the hippocamus^27,37^. Thus, the metabolic consequences of nPM exposure we observe could be due, at least in part, to effects in other parts of the brain. For example, nPM-exposed mice exhibited increased food intake by consuming more calories per eating event but not because of increased numbers of eating events. Furthermore, this disrupted eating pattern occurred only during the light cycle, a period during which mice normally exhibit lower overall food consumption^38^. These observations suggest that nPM may alter pathways in the hypothalamus that regulate food intake. This notion is supported by nPM-exposed mice having decreased hypothalamic expression of *Leprb*, which mediates the effects of leptin on satiety^39^. By contrast, expression of *Agrp* and *Npy* was opposite to what would be expected given the increased food intake in nPM-exposed mice. However, the relationship between hypothalamic expression of appetite regulating genes and eating behavior is likely more complex since it is known that orexigenic gene expression is diurnally regulated^40^ and can be dysregulated during perinatal programming, particularly in the context of obesity^41^. Interestingly, genetic disruption of the circadian clock in mice results in abnormal diurnal feeding rhythms characterized by increased food intake during the light cycle and the development of obesity-related phenotypes^42^. Similarly, multiple epidemiological studies have reported increased prevalence of obesity and metabolic syndrome in night and shift workers^43^ but the mechanisms responsible for these associations are not well understood.

By designing our study to expose mice *in utero* and postnatally up until young adulthood, we attempted to model the chronic exposure that children and adolescents undergo. Given the emerging epidemiological evidence that TRAP exposure is adversely associated with obesity-related outcomes, our results may be particularly relevant in these vulnerable pediatric populations. Other important translational factors need to also be taken into consideration, including the window of development during which mice were exposed and the model TRAP used. Interestingly, previous studies demonstrated that DEP, another model TRAP, increased susceptibility to diet-induced body weight, cognitive, and neuroinflammatory changes in adulthood even though the mice were only exposed *in utero*^28,44,45^. By comparison, both short-term and long-term exposure to PM_2.5_ during only the postnatal period with or without high feeding promoted visceral fat accumulation, the development of glucose intolerance, and other related metabolic abnormalities without affecting body weight^20–22^. These observations suggest that either prenatal or postnatal exposure results in different metabolic outcomes or that different forms of TRAP have particulate-specific effects. Importantly, nearly all of the physiological and cognitive consequences of ambient air pollution exposure in these previous studies were only observed in male mice, regardless of whether PM_2.5_ or DEP was used, which is consistent with the sex-specific effects of nPM on body weight trajectories in our study. Thus, these data provide strong evidence that exposure to different forms of TRAP, including nPM, during various stages of development that are relevant to both children and adults can have adverse biological effects in multiple organ systems involved in obesity and metabolism. Moreover, these results also suggest that males in particular may be more susceptible to the effects of TRAP, regardless of the exposure window or type of pollutant. However, it is not known whether nPM exposure only during the postnatal period, alone or in combination with high feeding, disrupts metabolic homeostasis in both sexes similarly. Additional studies will be needed to answer these important questions as well as determining whether sexually dimorphic associations of TRAP exposure exist in humans with respect to metabolic outcomes, eating behavior, and other cognitive traits.

Our results should also be taken in the context of certain limitations. For example, the re-aerosolized nPM that we used is representative of ambient PM in size distribution, water soluble carbon species, nitrate, sulfate, ammonium, and redox-active metal species, but is depleted in water-insoluble carbon species, such as black carbon and polycyclic aromatic hydrocarbons^25^. Thus, any biological effects of these and other water-insoluble components would not be tested in our studies. Second, the concentration of nPM that we exposed mice to (343ug/m^3^) is higher than would be experienced by individuals under typical real-world conditions and potentially not directly translatable to the average human population. Third, the power to detect statistically significant differences may have been hindered by the relatively small number of animals in some experimental groups, which could be addressed by having larger sample sizes in follow up studies. Finally, although ambient PM exposure has been shown to have indirect biological effects by promoting systemic inflammatory responses that could in turn affect tissue function^23,26^, nPM could potentially also have direct effects since particles have been detected in tissue. For example, in neuronal tissues, potentially via translocation along the olfactory nerve^46,47^. PM has also been postulated to enter the body by as a result of ingestion^46^, and it is not known whether inhalation exposure would result in localization of ambient PM to adipose tissue. Determining whether the metabolic disturbances we observed are due to indirect or direct effects of nPM will need to be addressed in future studies.

In summary, we demonstrate that exposure to nPM from gestation to early adulthood impairs metabolic homeostasis in male mice. While previous animal studies have yielded similar findings with respect to other model TRAPs, such as PM_2.5_ and DEP, our results are the first to directly implicate particles in the ultrafine range as having obesity-related effects. Taken together, these results add to the growing body of evidence that exposure to multiple forms of TRAP is associated with development of obesity and diabetes-related abnormalities in both mice and humans, and indicate that some of pathways mediating the effects of nPM on metabolism could be related to appetite regulation. Understanding the causal nature of these increasingly recognized relationships and their underlying biological mechanisms will need to be addressed in future studies.

## Materials and Methods

The data that supports the findings of this study are available from the corresponding author upon request.

### Animal husbandry

Male and female C57BL/6 J mice were purchased from the Jackson Laboratories (Bar Harbor, Maine) and bred in-house for this study. Per group, 6–9 mice from these mating pairs were used, housed 4–5 per cage at 25 °C on a 12 hr dark/12 hr light cycle and maintained on a chow diet (Purina #5053). All procedures were approved by the Institutional Animal Research Committees of the University of Southern California and Children’s Hospital Los Angeles, and all methods were performed in accordance with the relevant guidelines and regulations.

### Particulate collection and extraction

Ambient nPM were collected on Zeflour PTFE filters (Pall Life Sciences, Ann Arbor, MI) by means of a High-Volume Ultrafine Particle (HVUP) Sampler^48^ at 400 L/min flow rate at the Particle Instrumentation Unit (PIU) of USC within 150 m downwind of a major freeway (Interstate 110). These aerosols represent a mix of fresh ambient PM mostly from vehicular traffic^49^ and have been extensively characterized and described in previous studies^25^. Briefly, the majority of these particles are under 100 nm but in order to capture a wider and more full range of primary emission particles than what is traditionally referred to as ultrafine particulate matter (PM_0.1_)^50^, we collected and used particles with aerodynamic diameter ≤200 nm. The mass concentration of nPM was determined based on weighing of the filters before and after collection under controlled temperature (22–24 °C) and relative humidity (40–50%). Filter-deposited dried nPM were eluted by sonication into deionized water, following a previously described approach^34,35^. Frozen stocks (150 μg/ml) were kept at −20 °C and re-aerosolized with a HOPE nebulizer (B&B Medical Technologies, Carlsbad CA). The size distribution of the re-aerosolized nPM was comparable to typical ambient aerosols^51^ and the chemical composition of ions (NH_4_^+^, NO_3_^−^, SO_4_^2−^) and water soluble organic compounds was similar to ambient air at the collection site^25^. However, the re-aerosolized nPM was depleted in water insoluble species, including black carbon and polycyclic aromatic hydrocarbons^25^. Our prior studies have shown nPM collected in this manner to retain chemical stability for >30 days, including long-lived free radicals^24,25^, and have trace endotoxin levels.

### Exposure protocol

Female mice were checked for vaginal plugs 12 hours after being placed in a mating pair with males, then randomly selected for exposure to re-aerosolized nPM (at mass concentration of 343 μg/m^3^) for 5 hrs/day, 3 days (MWF)/week spanning *in utero* period. Control animals were exposed in a parallel exposure system to HEPA-filtered ambient air in which particle numbers were below the level of detection. After birth, pups continued to receive exposure to nPM or filtered air while suckling in their home cages until weaning. At weaning, mice of both sexes from multiple litters within each exposure group were randomly selected for group housing. Mice continued to receive exposure until 15 weeks of age (total of 18 weeks exposure), after which mice were euthanized at 18 weeks for tissue analysis. Mass concentration of the re-aerosolized nPM exposure stream was measured by gravimetric analysis of filters parallel to the exposure stream. The number and mass-based concentrations of the inlet aerosol were monitored throughout the exposure period using the condensation particle counter (TSI Inc., Shoreview MN) and the DustTrak™ II Aerosol Monitor 8532 (TSI Inc., Shoreview MN), respectively.

### Body weight and composition

Body weights were measured on a weekly basis starting at weaning (4 weeks of age) until 15 weeks. Whole body fat, fluids, and lean tissue mass were determined by NMR (Echo MRI, Houston, TX) according to the manufacturer’s recommendations. Adiposity was calculated by dividing total fat mass by total body weight.

### Food intake, locomotor activity, and energy expenditure

Mice were acclimated to individual housing for 3 days and placed into TSE Phenomaster/Labmaster Metabolic Home Cages (TSE Systems, Chesterfield, MO). Real-time monitoring of food intake was assessed from spill proof bottles attached to high precision weighing sensors. Food intake per 10 minute intervals, locomotion, energy expenditure, and respiration were measured continuously over 72 hours. Daily caloric intake, number of eating events, and average consumption per event was calculated. Spontaneous locomotor activity on the x and y axis was measured every minute with infrared light beams. For energy expenditure and respiration O_2_ and CO_2_ concentrations were measured in each cage every 10 mins and the respiratory exchange ratio (RER) was calculated as VCO_2_/VO_2_. Energy expenditure was calculated using the Weir Equation (heat = kcal/hr = (3.815 + 1.232 × RER) × (VO_2_)). Values were normalized to body weight.

### Behavior testing

All behavioral testing was performed on mice between 12–15 weeks of age.

#### Open field exploration

Mice were placed in a black Plexiglass box (40 × 45 × 35 cm) for one 30-min testing period. Movement was recorded and analyzed by Noldus software. Velocity, total distance traveled, and time spent in periphery versus center were analyzed. Center area was defined by a 30 × 30 cm area in the middle of the testing chamber.

#### Rotarod

Motor function was assessed by rotating rod testing (Rotarod #3375-M5, TSE Systems, Homburg Germany). The time before falling was measured under 4 RPM, 16 RPM, and accelerating protocols, with a maximum trial time of 120 seconds. Each mouse underwent three trials for each protocol, with each trial separated by 30 minutes and each protocol tested on a separate day.

#### Spontaneous alternations in the Y-maze

To test working memory, an apparatus consisting of three equivalent black Plexiglass arms (15 × 8 × 10 cm) separated by equal angles was used. Mice were placed in one arm and allowed free exploration for 10 min. The sequence and entries into each arm were recorded and percent alternation was determined from successive consecutive entries to the three different arms, divided by the total number of transitions.

#### Novel object exploration and recognition

To test recognition memory, mice were habituated to Makrolon cages for 15 minutes. The day after, animals explored two novel black plastic cylinders (8 cm tall × 3.5 cm in diameter) affixed to the floor and symmetrically placed 6 cm from the two nearest walls. Mice were placed in a corner, facing the center and at equal distance from the objects. Their start position was rotated and counterbalanced throughout the test. Long term memory was tested twenty-four hours later, by replacing one cylinder with a novel plastic rectangular block (6 cm tall × 3 cm × 3 cm), placed in a counterbalanced fashion to avoid experimental bias. Analysis included the number and total duration of exploratory approaches between objects. Exploration was defined as sniffing or touching the objects with the snout; sitting on the object was not considered exploration. Novel object exploration index was calculated by dividing exploration of the novel object by total exploration.

#### Elevated plus

Mice were tested for anxiety behavior on the elevated plus maze (67 cm arm length, 5.5 cm arm width, 39.5 cm height). Mice were allowed free exploration for 5 minutes, with movement recorded and analyzed by Noldus software. Total movement, time spent in open arms, and number of arm entries was measured.

### Intraperitoneal glucose tolerance tests

Intraperitoneal glucose tolerance tests (IPGTTs) were carried out on mice that were fasted for 5 hours, as described previously^52^. A baseline blood sample (0 timepoint) was obtained through the tail vein prior to mice being injected with 1 g/kg body weight of glucose (10% wt/vol in sterile H_2_O) into the peritoneal cavity. Plasma glucose levels were measured from tail vein blood samples from conscious mice at 15, 30, 60, 90 and 120 minutes post injection. Glucose levels were determined using a Freestyle Lite glucometer (Abbott Diabetes Care, Alameda, California).

### Plasma measurements

Blood was obtained at euthanization using cardiac puncture for measurement of metabolic parameters in plasma. Insulin and leptin levels were measured in duplicate using MILLIPLEX MAP Mouse Adipokine Magnetic Bead Panel (Billerica, MA). Enzymatic assays for total cholesterol and triglyceride levels were performed as described previously^53^. Combined very low-density lipoprotein (VLDL) cholesterol and LDL cholesterol levels were calculated by subtracting high density lipoprotein (HDL) cholesterol from total plasma cholesterol levels.

### Determination of hepatic lipid content

Hepatic lipid content was measured from liver homogenates as described previously^54^. Briefly, frozen liver samples (~50 mg) were homogenized in 1.5 ml of 40 mM Tris-HCl buffer (pH 7.4) on ice and centrifuged at 12,000 rpm for 15 minutes at 4 °C. Cholesterol levels were measured using the Infinity Cholesterol Reagent (Thermo Scientific, Middletown, VA) and triglyceride levels were measured by a colorimetric assay (Cayman Chemicals, Ann Arbor, MI). Protein concentration was determined using the BCA assay kit (Thermo Scientific, Middletown, VA).

### Determination of neurotransmitters levels

Cortex samples were homogenized in a buffer containing 0.1 M TCA, 0.01 M sodium acetate, 0.0001 M EDTA, 5 ng/ml isoproterenol (as an internal standard) and 10.5% methanol (pH 3.8). Protein concentration was determined by a BCA Protein Assay Kit (Thermo Scientific), after which the samples were centrifuged at 10,000 g for 20 minutes. For each sample, 10 μl of the supernatant was diluted with 70 μl of borate buffer to which 20 μl aliquots of 6-aminoquinol-N-hydroxysuccinimidyl carbamate were added to form the fluorescent derivatives. After incubation at 37 °C for 10 minutes, concentrations of neurotransmitters and their metabolites were determined by HPLC on a Waters 2707 Autosampler instrument (Waters Corporation, Milford, MA), according to the manufacturer’s protocols. Data acquisition was managed by Empower 2 software (Waters Corporation, Milford, MA).

### Real-time gene expression

At the time of euthanization, subcutaneous and epididymal adipose depots and the hypothalamus were harvested and in snap frozen in liquid nitrogen. Total RNA was extracted using RNeasy Mini kits (Qiagen, Valencia, CA) and cDNA was prepared from 500 ng of total RNA using Archive Reverse Transcription kits (Life Technologies, Gaithersburg, MD) according to the manufacturer’s protocols. Real-time gene expression reactions were carried out in quadruplicate with SYBR green assays on the Fluidigm System (South San Francisco, CA). Transcript levels for each sample were calculated relative to a tissue-specific calibrator comprised of a pool of all samples. Each sample was normalized to *Ppia* or *Gapdh*, as endogenous controls, and the replicates were averaged to determine differences between control and nPM-exposed animals.

### Flow cytometry

Fresh samples of adipose tissue was weighed and digested with 200 U/ml Type-IV-collagenase (Worthington Biochemical Corporation, Lakewood, NJ) at 37 °C for one hour and passed through a 70 µm cell strainer (Corning). Cells from the stromal vascular fraction were stained with antibodies to the following surface markers: CD45, CD4, CD25, CD127, ST2, Lineage (CD3ε, CD11c, CD11b, B220, Ter-119, Gr-1, FcεRIα, and γδ T-cell receptor) (eBioscience, San Diego, CA). Thereafter, cells were fixed, permeabilized and stained for intracellular FoxP3 expression (eBioscience, San Diego, CA). T-cell subsets and type-2 innate lymphoid cells (ILC2s) were then isolated using an 8-color FACS Aria III cell sorter and a FACS Diva instrument for data collection (BD Bioscience, San Jose, CA). The following gating strategies were used: CD45^+^CD4^+^CD25^+^FOXP3^+^ to isolate regulatory T-cells (Tregs); CD45^+^CD4^+^CD25^+^ CD127^high^FOXP3^−^ for T effector cells (Teffs); and Lin^−^CD45^+^CD127^+^ST2^+^ for ILC2s. Immune cell subsets were quantified with Flowjo X software (Ashland, OR), which were back calculated to total cell counts and normalized to adipose tissue weight, as previously described^55^.

### Statistical analyses

Differences in measured variables between control and nPM-exposed mice were determined by Student’s *t*-test (PRISM v6.01). Values are expressed as mean ± SE and differences were considered statistically significant at p < 0.05.

## Supplementary information

Supplemental Information
