# Supplementary material for: Exposure to Nanoscale Particulate Matter from Gestation to Adulthood Impairs Metabolic Homeostasis in Mice
Source: Sci Rep. 2019 Feb 12;9:1816. doi: 10.1038/s41598-018-37704-2 (PMC6372675; doi:10.1038/s41598-018-37704-2)
Supplement: Supplementary file 1 — Supplemental Information [file 41598_2018_37704_MOESM1_ESM.pdf]

Supplementary Data

Exposure to Nanoscale Particulate Matter from Gestation to Adulthood Impairs Metabolic Homeostasis in Mice

Nicholas C. Woodward[1,2], Amanda L. Crow[1], Yang Zhang[1], Sam Epstein[2], Jaana Hartiala[1], Richard Johnson[2], Heidi Kocalis[3,4], Arian Saffari[5], Ishwarya Sankaranarayanan[6], Omid Akbari[6], Gajalakshmi Ramanathan[7,8], Jesus A. Araujo[7,8], Caleb E. Finch[2], Sebastien G. Bouret[3,4], Constantinos Sioutas[5], Todd E. Morgan[2], and Hooman Allayee[1]

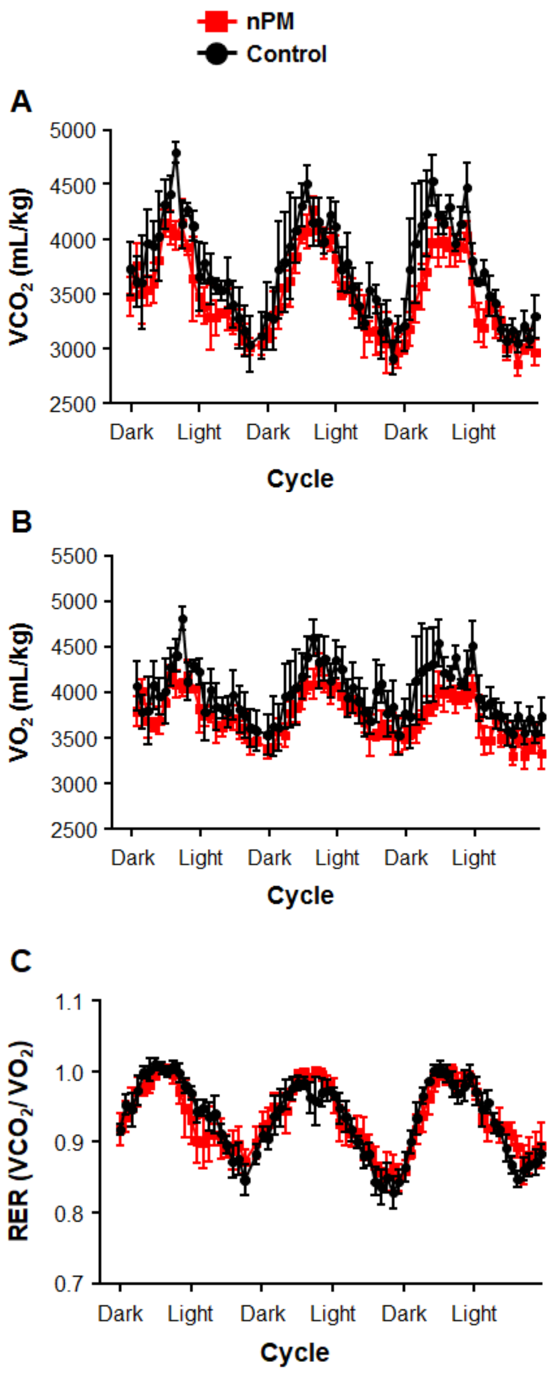

Supplementary Figure 1. Effect of nPM exposure on metabolic rate. Compared to the control group, mice exposed to nPM did not exhibit differences in CO_2 production (VCO_2) (A), O_2 consumption (VO_2) (B), or the respiratory exchange ratio (RER) (C). Data are shown as mean ± SE from 6-7 mice in each exposure group.

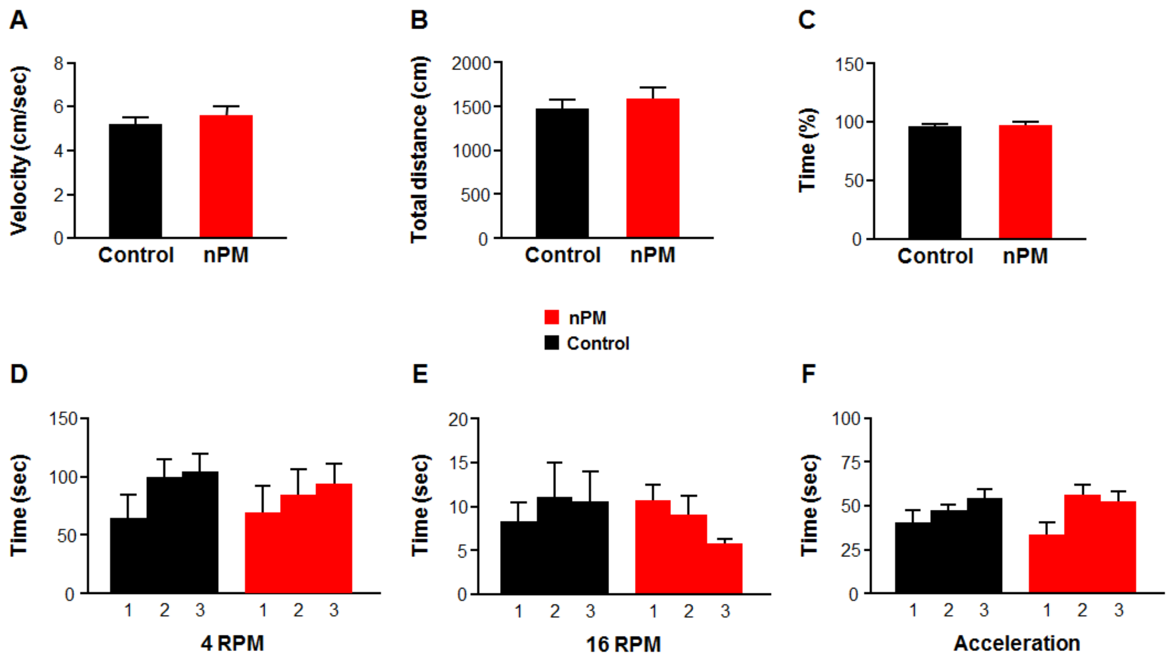

Supplementary Figure 2. Effect of nPM exposure on exploratory behavior and overall motor function. Open field tests did not reveal differences in velocity (A), total distance traveled (B), or time spent in the periphery (C) between nPM-exposed and control mice. Physical performance of nPM-exposed mice during three trials at 4 RPM (D), 16 RPM (E), or acceleration (F) rotorod protocols was also not significantly different than control animals. Data are shown as mean ± SE from 6-7 mice in each exposure group.

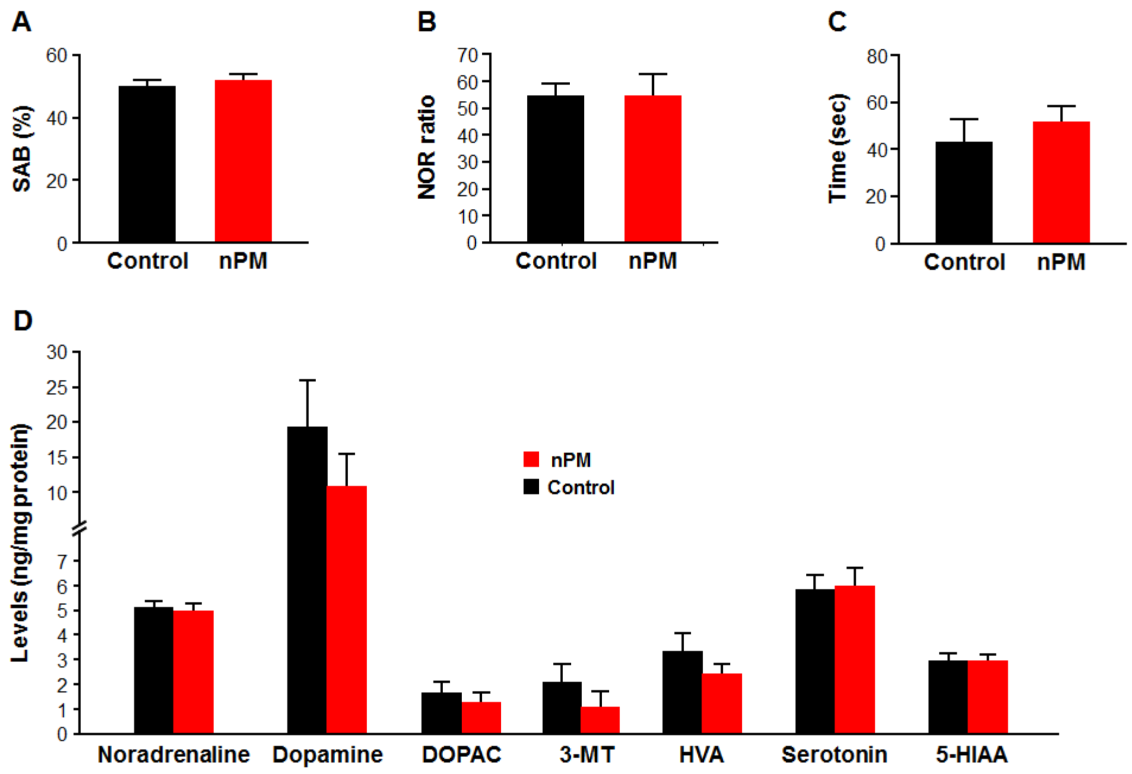

Supplementary Figure 3. Effect of nPM exposure on behavioral traits and brain neurotransmitter levels. Mice exposed to nPM did not exhibit working and recognition memory deficits, as determined by spontaneous alternation behavior (SAB) in Y-maze and novel object recognition (NOR) tests (A and B, respectively), or increased anxiety based on the amount of time spent in the open arm during an elevated plus maze test (C). Levels of neurotransmitters and their metabolites, such as 3,4-dihydroxyphenylacetic acid (DOPAC), 3-methoxytyramine (3-MT), homovanillic acid (HVA), and 5-hydroxyindoleacetic acid (5-HIAA), were not significantly different in the cortex of nPM-exposed mice compared to controls (D). Data are shown as mean ± SE from 6-7 mice in each exposure group.
